# Anaphylactic reactions during oral food challenge test in pediatric patients

**DOI:** 10.1186/2045-7022-3-S3-P121

**Published:** 2013-07-25

**Authors:** AF Gushken, LA Watanabe, CL Beck, APBM Castro, GH Yonamine, AC Pastorino, CMA Jacob

**Affiliations:** 1 Department of Pediatrics, Child’s Institute, São Paulo, Brazil; 2 Divisão de Nutrição, Child´s Institute, São Paulo, Brazil

## Background

The prevalence of Food Allergy (FA) is increasing and the correct diagnosis is mandatory among patients with adverse reactions to foods. The oral food challenge tests (OFCT) remain the better tests for FA diagnosis and its usage is essential to avoid misdiagnosis. As severe reactions may occur, staff must be prepared to manage life- threatening situations. The aim of this study was to describe the anaphylactic reactions associated to OFCT in pediatric patients.

## Methods

During the last ten years, 205 OFCT were done in 155 patients for FA diagnosis or to confirm the development of tolerance. Among all tests: 46 were open challenge (median age: 23 mo) and 159 blind challenges (median age: 52 mo) being 3 of them simple blind and 156 double blind placebo controlled challenge test (DBPCCT) Milk was the most frequent food evaluated (n=194), followed by egg(n=9) and wheat (n=2). Anaphylaxis criteria were based on Sampson et al, 2006. All the patients’ guardians signed an informed consent form.

## Results

From 205 OFCT performed, 17 (8.3%) of them resulted in anaphylaxis, being 13 during DBPCCT. Median age of anaphylactic patients was 6.12 years (range1.28-14.30y). Among these patients, 12 referred previous anaphylaxis. During the OFTC with milk, clinical initial symptoms were runny and itchy nose, cough, itchy throat and redness of face. During anaphylaxis episodes, the cutaneous symptoms were the most frequent (100%) followed by respiratory (70.5%) and gastrointestinal (52.9%) ones. The clinical manifestations started with variable milk volumes (median 5 ml-from 1 to101ml) and anaphylaxis was diagnosed with median volume of 16 ml (1-101 ml). Epinephrine IM was administered in 10 patients at outer thigh in recommended doses and the others recovery with antihistamines or bronchodilators. Biphasic reaction was not observed in any patients. All of them showed complete recovery, without necessity of intensive care.

## Conclusion

In this study, mild respiratory symptoms were observed immediately before the anaphylaxis episode although not reported in the clinical history. The prompt recognizing and approach to anaphylaxis avoided more severe complications, being the OFCT safe and still considered an important tool in FA diagnosis.

## Disclosure of interest

None declared.

